# Effect of Fibre Supplementation on Body Weight and Composition, Frequency of Eating and Dietary Choice in Overweight Individuals

**DOI:** 10.3390/nu9020149

**Published:** 2017-02-16

**Authors:** Vicky A. Solah, Deborah A. Kerr, Wendy J. Hunt, Stuart K. Johnson, Carol J. Boushey, Edward J. Delp, Xingqiong Meng, Roland J. Gahler, Anthony P. James, Aqif S. Mukhtar, Haelee K. Fenton, Simon Wood

**Affiliations:** 1 School of Public Health, Faculty of Health Sciences, Curtin University, Perth WA 6845, Australia; d.kerr@curtin.edu.au (D.A.K.); w.hunt@curtin.edu.au (W.J.H.); s.johnson@curtin.edu.au (S.K.J.); T.P.James@curtin.edu.au (A.P.J.); Aqif.Mukhtar@curtin.edu.au (A.S.M.); h.fenton@curtin.edu.au (H.K.F.); simonwood@shaw.ca (S.W.); 2 Epidemiology Program, University of Hawaii Cancer Center, Honolulu, HI 96813, USA; cjboushey@cc.hawaii.edu; 3 Video and Image Processing Laboratory, School of Electrical and Computer Engineering, Purdue University, West Lafayette, IN 47907, USA; ace@ecn.purdue.edu; 4 Flinders Centre for Innovation in Cancer, School of Medicine, Flinders University, Adelaide 5001, Australia; rosie.meng@flinders.edu.au; 5 Factors Group R & D, Burnaby, BC V3N4S9, Canada; rgahler@naturalfactors.com; 6 Curtin Health Innovation Research Institute, Faculty of Health Sciences, Curtin University, Perth WA 6845, Australia; 7 Centre for Population Health Research, Faculty of Health Sciences, Curtin University, Perth WA 6845, Australia; 8 InovoBiologic Inc., Calgary, AB Y2N4Y7, Canada; 9 Food, Nutrition and Health Program, University of British Columbia, Vancouver, BC V6T1Z4, Canada

**Keywords:** fibre, weight, waist circumference, frequency, eating, PolyGlycopleX^®^ (PGX)

## Abstract

Fibre supplementation can potentially reduce energy intake and contribute to weight loss. The mechanism may be reduced frequency of eating, resulting in reduced food consumption. The objective of this research was to determine the effectiveness of fibre supplementation with PolyGlycopleX^®^ (PGX^®^), on body weight and composition, frequency of eating and dietary intake in 118 overweight adults. In a three-arm, parallel, blind, randomised controlled trial participants were randomised to one of three groups; 4.5 g PGX as softgels (PGXS), 5 g PGX granules (PGXG) or 5 g rice flour (RF) control. Prior to supplementation and at 12 weeks, participants captured before and after images of all food and beverages consumed within 4 days using a mobile food record app (mFR). The mFR images were analysed for food group serving sizes and number of eating occasions. In the PGXG group, intention-to-treat analysis showed there was a significant reduction in waist circumference (2.5 cm; *p* = 0.003). Subgroup analysis showed that PGXG supplementation at the recommended dose resulted in a reduction in body weight (−1.4 ± 0.10 kg, *p* < 0.01), body mass index (BMI) reduction (−0.5 ± 0.10, *p* < 0.01), reduced number of eating occasions (−1.4 ± 1.2, *p* < 0.01) and a reduced intake of grain food (−1.52 ± 1.84 serves, *p* = 0.019). PGXG at the recommended dose resulted in a reduction in weight and BMI which was significantly greater than that for RF (*p* = 0.001). These results demonstrate the potential benefits of PGX fibre in controlling frequency of eating and in weight loss.

## 1. Introduction

The burden of chronic disease in many countries is increasing concomitant with the percentage of the population with a high body mass index (BMI) [1]. Providing tools to improve the healthiness of the food environment and assisting people to make healthy food choices are important in the prevention of excessive weight gain [1]. 

Dietary pattern evidence has shown that higher dietary quality i.e. consumption of mainly whole grains, vegetables, fruit, nuts, legumes, seafood, plant protein and low-fat dairy leads to marked reductions in all-cause, cardiovascular disease and cancer mortality [2,3]. Similarly, a healthy dietary pattern which includes consumption of fruits, vegetables, grains, combined with a lower intake of sweets, red meat and processed meat, lowers the risk of developing colorectal cancer [4].

Evidence supports the role of dietary fibre in improving metabolic health. A higher intake of fibre is associated with increased satiety and reduced energy intake and therefore may be important in obesity management [5,6,7,8,9,10,11]. 

Total fibre is the sum of both dietary fibre (complete, intrinsic non-digestible plant carbohydrates, lignin) and functional fibres (non-digestible carbohydrates that have been isolated but still have beneficial physiological effects) [12]. What constitutes a ‘beneficial physiological effect’ and the level of evidence required for this to be substantiated remains unspecified [13]. Solubility or the ability of a fibre to dissolve in water will affect water holding capacity, viscosity and fermentability [11]. The properties of fibre that are associated with appetite, energy intake and body weight include solubility [11]. Solubility remains a common classification technique, although it has been suggested that fibres should be categorised according to functional properties such as viscosity and fermentability [14], since not all fibre is equal in delivering a beneficial physiological effect.

PolyGlycopleX (PGX) is a commercial functional fibre complex, manufactured by a proprietary process (EnviroSimplex^®^) from three dietary fibres: konjac glucomannan, sodium alginate, and xanthan gum [9]. PGX is a soluble viscous non-starch polysaccharide complex that has been identified as contributing to improved satiety, lipidaemia and glycaemia [8,9,10,15,16].

In the modern food environment both portion size and frequency of eating have increased in the population [17]. Mattes [17] has suggested that increased frequency of eating is a major factor in weight gain and is linked to the increased energy intake and rising BMI trends. In addition to frequency of eating, the types and amount of food and beverages consumed at an eating occasion may influence energy intake. Aljuraiban et al. [18] suggest that modifying eating behaviour through more frequent meals of low energy density and high nutrient quality may be an important approach to controlling obesity. 

A challenge for understanding the contribution of dietary factors is our ability to measure diet. This is even more difficult in overweight and obese participants who are more prone to misreporting or underreporting their energy intake [19,20]. In addition, details such as time of eating is important in examining eating frequency and are difficult to accurately capture with paper-based methods. Advancement in technology has made available new image-based food recording systems such as those using a mobile food record app (mFR) [21,22,23,24,25]. Among the advantages of mFRs is the provision of real-time data capture which allows for the extraction of information from the images on the timing and location of the eating occasion, without relying on individuals to report these details [22,23]. Thus the mFR app allows for more accurate capture of time of eating while reducing the burden to the participant of reporting food consumption [23,24]. A unique aspect of this study was the use of the mFR to capture frequency of eating and dietary intake. 

The objective of this research was to determine the effectiveness of diet supplementation with the viscous and gel-forming fibre, PolyGlycopleX (PGX), on body weight and composition and to determine if frequency of eating and diet can explain subsequent changes in 118 overweight adults.

## 2. Materials and Methods

### 2.1. Study Design 

**Figure 1:**
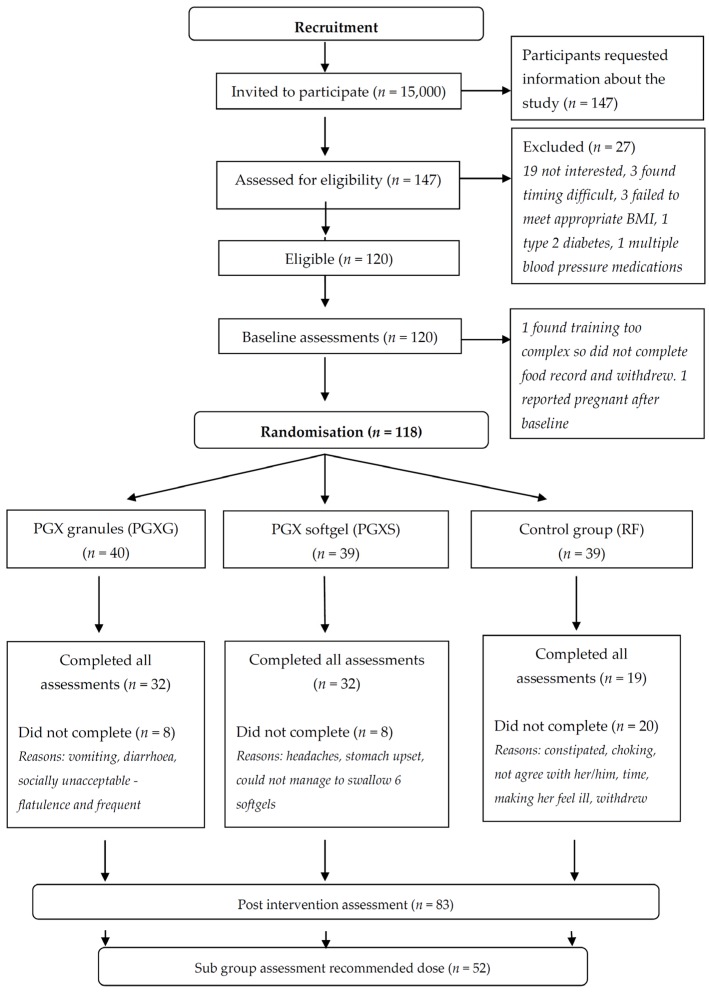
Participant flow diagram. RF: rice flour.

A three-arm, parallel, blind, randomised control trial was conducted to determine whether PGX supplementation would promote weight loss and reduce waist circumference and BMI, change dietary patterns and reduce the frequency of eating or number of eating occasions. Participants (118 in total) were divided between three groups, 4.5 g PGX as softgels (PGXS), 5 g PGX granules (PGXG) and 5 g rice flour (RF) control, (Figure 1) and were assessed at baseline and 12 weeks. This trial was registered with the Australian New Zealand Clinical Trials Registry (reference ACTRN12614000701628). 

The food images sent via the mFR were stored by a participant identification number (ID) only. No personal information was stored with the images. The research was conducted in accordance with the principles proposed by the Australian Association for Research in Education (AARE), the Australian Vice-Chancellor’s Committee (AVCC) and the National Health and Medical Research Council (NHMRC). The study was conducted in accordance with the Declaration of Helsinki and ethics approval was granted by the Human Research Ethics Committee, Curtin University (reference HR170/2014).

### 2.2. Study Participants

Participants, aged 25–70 years and with BMI 25–35 kg/m^2^ were recruited through advertisements on Curtin University radio, as well as email communication systems. Individuals that expressed interest were screened for eligibility by completing a screening questionnaire. Participants were excluded if they were: (a) pregnant; (b) unable to complete the 12-week study; (c) undertaking extreme forms of exercise or dieting; (d) unable to attend the study centre; or (e) had an allergy to any food ingredient used in the study; (f) had previous or current renal, liver or respiratory failure; (g) had previous gastric or weight-loss surgery; (h) had any malabsorption conditions or (i) had current or recent dietary fibre supplementation. The participant flow diagram (Figure 1) lists the reasons for exclusion. Once assessed as eligible, further details of the study were provided and informed consent obtained.

### 2.3. Baseline Assessments 

Participants who met the selection criteria had height, weight and waist circumference anthropometric measurements taken at baseline. A portable stadiometer was used to measure height; weight was measured using a pre-calibrated digital scale and waist was measured as described by Norton and Olds [26]. To electronically record food consumption, iPods with mFR application installed were given to the participants, who were asked to keep a 4-day mFR at baseline and during the twelfth week of the study. 

Before beginning their baseline image-based 4-day food record, all participants received a 30-minute interactive training session by a single researcher who conducted individual or small group training sessions using PowerPoint slides on how to connect the iPod to the Wi-Fi and how to use the mFR app. Training sessions were held at Curtin University in a room with Wi-Fi access. The inclusion of a fiducial marker (a checkerboard pattern of known shape, size and colour) in all food record images gave a known reference of dimension and markings to assist with food identification and portion size estimation [25]. During training, participants were able to practice taking before and after images using food models. Collected food record images were automatically uploaded from the iPod touch, when in Wi-Fi range. The iPods were coded with the participant ID ensuring each image was tagged with the participant ID, date and time of eating occasion. Participant ID was used to identify the images on the server. The server was accessible by researchers only via password.

Prior to the commencement of supplementation and during the twelfth week of the study, participants took before and after images of all foods and drinks (excluding water) consumed over four consecutive days. Food consumption during the entire study was ad libitum. 

### 2.4. PGX Supplementation 

The PGXS, PGXG and RF control supplements were provided to participants in a carry bag containing a 12-week supply labelled with a three-digit code. PGXG and the control were provided as 5 g individual doses in identical foil sachets and PGXS was provided in plain white jars each containing one month’s supply. Research staff were blinded to the treatment allocation until all analyses were completed.

In Arm 1 (PGXS) instructions were provided in writing and verbally to the participants: “Take 1–2 softgels three times a day in week 1, 2–4 softgels three times a day in week 2 and 4–6 softgels, three times a day in week 3 to week 12”. The recommended dose was four (4) to six (6) softgels containing 0.64 g fibre each, three times a day. This represented a supplement of dietary of between 7.6–11.4 g/day. Participants were also asked to consume 500 mL water with every softgel dose. Participants in Arm 2 were instructed to consume 5 g of PGXG containing 4.4 g fibre provided in a single dose foil sachet taken three times a day just before or with meals over the 12-week intervention period. This represented a supplement of dietary fibre of 12.2 g/day. Those in Arm 3 were provided with 5 g of RF containing 4 g fibre in the same dose format as the PGXG, representing 12 g fibre/day. RF was selected due to its neutral taste and hypoallergenicity, and it has a similar dietary fibre content, energy, colour and texture to PGX [7]. The recommended dose was 1 sachet, three times per day. Directions were “Stir 1 sachet into your meal or in a drink and consume immediately. You must consume 500mL of water each time you take a sachet.” Participants were also advised that “If you have any discomfort, reduce the dose to 1 sachet a day for week 1, 2 sachets for week 2 and 3 sachets week 3 to week 12. Contact the researcher to discuss any issues.” Participants were informed of the importance of consuming water with the supplement as well as the possible gastrointestinal effects of fibre such as diarrhoea, bloating and flatulence as described in research by Kacinik et al. [7]. All participants were instructed to record their daily sachet or softgel intake and report in person to the researcher at the end of week 12 of the intervention.

### 2.5. Post-Intervention Assessments

At the end of the 12-week intervention period, measurement of participants’ height, weight and waist circumference were repeated, along with the 4-day mFR being repeated during week 12. At the end of the 12-week intervention, during the final meeting participants reported the number of doses to the interviewer and data were recorded in participant files.

### 2.6. Dietary Analysis

A researcher reviewed the 4-day food record images and as needed, confirmed the content of images with participants. Eating occasions were defined as all food and beverages (except water) and were taken from the image metadata and any additional notes supplied by the participant. Eating occasions were categorised as beverage only, food only, single item, food and beverage or fibre only. The images of fibre also allowed dose of fibre taken to be determined. Analysis was conducted to measure any change in eating occasions from baseline to study week 12. 

Images were analysed and eating occasions, types of foods and serving sizes were entered into a database specifically designed to capture the number of eating occasions and food groups according to the Australian Guide to Healthy Eating food group serves (vegetables, fruit, grain (cereal) foods, mostly wholegrain and/or high cereal fibre varieties, lean meats and poultry, fish, eggs, tofu, nuts and seeds and legumes/beans, milk, yoghurt cheese and/or alternatives, mostly reduced fat) (NHMRC 2013) plus junk food (Table 1).

The primary outcome variables measured at baseline and at the end of the intervention were: changes in weight, waist circumference and BMI; eating occasions and foods consumed each day classified as serves of junk food (energy-dense nutrient poor), grain (cereal), meat, dairy, fruits and vegetables for the three intervention groups. Within each intervention group, participants were further categorised into fibre dose compliance level and number of eating occasions and the primary outcomes for this subgroup were also evaluated.

### 2.7. Statistical Analysis

Both intention-to-treat analysis and per-protocol analyses were performed and reported. The distribution of all outcome variables, weight, waist and BMI were checked by construction of histograms to check normality. A mixed effect model with clustering of participants’ ID and robust variance-covariance estimation were used to assess outcome variables. For the intention-to-treat analysis, all randomised participants were included and all available data at each study time point were used. For per-protocol analysis, only those who completed the study at week 12 were included and missing values were not imputed. Further analysis of subgroups by actual dose consumed was performed and reported. All tests were two tailed and a *p* value < 0.05 was regarded as statistically significant. All analyses were performed using Stata MP 14.1 (Stata Corp., College Station, TX, USA).

## 3. Results 

### 3.1. Baseline Characteristics

#### 3.1.1. Intention-to-Treat Analysis

Baseline measurements showing of all recruited participant characteristics according to treatment group are shown in Table 2. There were 92 females and 28 males, evenly distributed across the three treatment groups. In total 83 of the 118 participants (63% retention) completed the 12-week study. Greater attrition was observed in the first two weeks of the supplementation for participants who reported stomach upsets and diarrhoea after PGXG consumption (attrition = 6); who reported diarrhoea, headaches, difficulty swallowing recommended PGXS dose (attrition = 6) and during the first six weeks of supplementation in the RF group (attrition = 15) due to constipation and feeling ill. 

Baseline data (Table 2) shows the characteristics of study participants randomised (*n* = 118) and shows that at baseline there were no significant differences (*p* > 0.05) in age, height, weight, waist circumference, BMI and food group servings across three groups. At baseline PGXS participants had similar baseline frequency of eating (number of eating occasions) to the PGXG and RF participants but those allocated to the PGXG intervention had a significantly greater number of eating occasions per day than those allocated to the RF intervention (*p* = 0.04) (Table 2).

#### 3.1.2. Per-Protocol Analysis

Baseline measurements according to treatment group showing participant characteristics of all who consumed the recommended dose of fibre supplements are shown in Table 3. Baseline data (Table 3) shows the characteristics of study participants (*n* = 54) and shows that at baseline there were no significant differences (*p* > 0.05) in weight, waist circumference and BMI across three groups. PGXG and RF intervention sub-group numbers of eating occasions per day were not significantly different to each other or to the baseline group but the PGXS subgroup number of eating occasions was 7.4 times per day (Table 3).

### 3.2. Effect of Intervention on Body Weight and Body Composition 

#### 3.2.1. Intention-to-Treat Analysis

Intention-to-treat analysis revealed a significant reduction in waist circumference at week 12 of minus 2.5cm (Confidence Interval = −3.9, −0.8, *p* = 0.003) for those in the PGXG group (Table 4). No effect was seen on waist circumference for the PGXS and RF groups (*p* > 0.05). There was no effect of the PGXS or PGXG interventions on weight and BMI.

#### 3.2.2. Per-Protocol Analysis

Per-protocol analyses of the subgroup who consumed the recommended dose of fibre supplements showed a significant weight loss and reduction in BMI in the PGXG intervention compared to baseline (Table 5). However, the PGXS subgroup did not show a significant weight change during the intervention period. Compared to effect of the intervention on weight seen in the RF subgroup, that of the PGXG subgroup was significantly greater (*p* = 0.001).

### 3.3. Number of Eating Occasions and Food Group Servings

**Figure 2:**
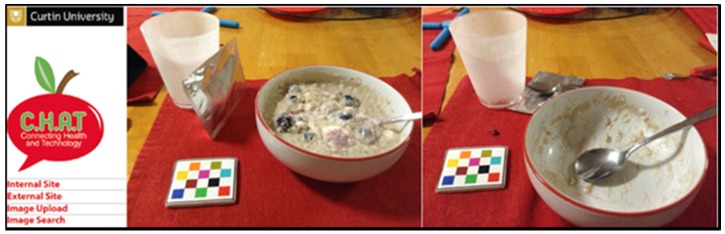
Before and after images of an eating occasion with foil sachet containing PGXG and fiducial marker captured with the mobile food record App on an iPod.

Collection of mFR images required participants to use the iPod provided by the study to record images before and after each eating occasion during the 4-day food record (Figure 2).

#### 3.3.1. Intention-to-Treat Analysis

Analysis was conducted to measure change in number of eating occasions from baseline to week 12 of the interventions. The number of eating occasions was significantly reduced in the PGXG group (*p* = 0.01) during the intervention (Table 4). No significant differences in number of eating occasions between baseline and week 12 were observed in the groups PGXS and RF or in food group servings in any of the intervention groups (Table 4) in the intention-to-treat analyses. 

#### 3.3.2. Per-Protocol Analysis

For the per-protocol analysis, the subgroup who consumed PGXG at the recommended dose of 2.5 to 3 times per day, significantly reduced their eating occasions by 1.4 ± 1.2 (CI −2.1, −0.6, *p* < 0.01) during the intervention period (Table 5). 

Likewise, the participants who consumed PGXG at the recommended dose significantly reduced their daily intake of fruit (−0.63 ± 0.57 serves, *p* = 0.02), dairy (−0.59 ± 0.50 serves, *p* = 0.01) and grain food (−1.52 ± 1.84 serves, *p* = 0.02) during the intervention (Table 5). Analysis of types of foods in the mFR showed that 1.6 to 2 serves of the grain food consumed at baseline were white bread for these participants. This consumption of white bread for the PGXG group also dropped significantly to 0.74 serves per day at 12 weeks of intervention.

### 3.4. Adverse Events

Adverse effects reported by the participants were mild and agreed with common reported reactions to increased fibre, for example those reported by Kacinik et al. (2011) [7] where for PGX supplementation, 30.9 % of participants reported diarrhoea, bloating and flatulence whereas for rice flour consumption 7.8% reported constipation.

## 4. Discussion

This 12 week randomised controlled study showed that when consumed at the recommended dose (per-protocol), the PGXG intervention gave a reduction in BMI and body weight, the number of eating occasions per day and consumption of servings of grain food.

The weight loss and BMI reduction observed for the PGXG per-protocol intervention is most likely a result of compliance to the recommended dose, resulting in 64% of participants who consumed PGXG at the recommended dose losing weight and reducing BMI. The average 1.4-kg weight loss found in our study of overweight adults for the PGX per-protocol intervention is in agreement with previous research by Lyon et al. [28] who found a weight loss in women of 1.6 kg with 12 weeks PGX supplementation. Pal et al. [29] reported obese adults on 12-week supplementation of both artificially sweetened and flavoured PGX and psyllium lost weight (1.6 kg and 1.1 kg respectively). 

On an individual basis, the highest weight loss in the PGXS group (intention-to-treat) was 4.4 kg, with a waist circumference decrease of 5.5 cm. However, in this group a weight gain of 6.3 kg and waist circumference increase was 9.6 cm was also recorded in one participant. This high variance in change in weight and waist circumference resulted in a non-significant weight loss for this group. Data collected by personal communication at the 6-week visit showed five participants in the PGXS group reporting that they expected PGXS to be a “magic bullet” for weight loss, which was an unexpected issue that requires consideration in future research. Protocol in future research may involve education of participants on using PGX to control appetite.

The significant reduction in waist circumference observed in the present study for PGXG intention-to-treat group of 2.5 cm was similar in magnitude to that reported in a study by Reimer et al. [8] where PGX consumption over 14 weeks resulted in a significant reduction of 1.96 cm in waist circumference. The significant reduction in BMI of 0.5 observed in the present study for PGXG per-protocol sub-group was in contrast to Reimer [8] who found no significant differences in BMI compared to baseline. 

The findings from our study that PGXS and PGXG consumption (intention-to-treat groups) did not affect weight is similar to research on PGX consumption by Kacinik et al. [7] who reported no significant differences in weight loss between PGX and the placebo groups in a study involving a low-calorie diet of 1000 kcal/day for both treatments. There was also no weight loss recorded in a three-week study by Reimer et al. [30] where PGX was pre-mixed with breakfast cereal and consumed with yogurt. In our study participants were not on an energy restricted diet nor were the PGX and RF doses mixed with other products.

The use of the mFR allowed our research to determine level of compliance to the recommended dose of the PGXG per-protocol group by reviewing the images collected. The mFR image analysis of the foods eaten, serving sizes and number of eating occasions per day enabled important information about the dietary pattern of the overweight participants to be collected. The reduced number of eating occasions found with the PGXG per-protocol subgroup, translated into reduced consumption of grain food of 1.52 serves (*p* = 0.019), mainly being a reduction in consumption of white bread. 

Research by Kerr et al. [23] reported a reduction in consumption of energy-dense nutrient-poor (EDNP) foods as a result of tailored dietary feedback using the mFR. In the present study there was a non-significant reduction in junk food consumption of 0.57 in the PGXG intention-to-treat group and 0.76 serves in the PGXG per-protocol group. Pollard et al. [31] also found overweight people were more likely than those who were a healthy weight to decrease their intake grain food when trying to lose weight, supporting the reduced grain food trend found in this research. Small reductions in conscious or mindful energy intake can improve weight gain [32] and the choice to reduce grain food intake found in this research may have been the contributor to weight loss. Although we observed a reduction in daily intake of fruit and dairy, which is not desirable based on dietary guidelines [27], the magnitude of this change was minor.

Data from the mFR showed participants in all groups, other than the PGXG per-protocol subgroup, appeared to make no changes to their eating pattern, as after 12 weeks of intervention, food group servings were not significantly different to baseline. This finding is supported by previous studies [32,33,34] where it was reported that humans tend to consume a consistent weight or volume of food from day to day. While participants reported feeling full in previous PGXS and PGXG research [9,10,35,36], most participants in this study did not appear to use appetite to change their eating patterns, except in the per-protocol PGXG group who consumed the recommended dose, most likely resulting reduced appetite and reduced food intake. 

Dietary feedback using the mFR indicated a reduction of 1 serve white bread, each day, which may have been a contributor to the reduction of 40 g in weight daily in the PGXG per-protocol group [27]. In a previous study, a reduction of 31.5 g carbohydrate per day was recorded at 12 weeks after PGX supplementation using a 3-day food and drink diary [29]. The 3-day food and drink diary does not provided details on type carbohydrates foods, whereas the mFR reports provided detail of types of foods and serving sizes. Dietary intake records collected using the mFR can reduce the bias found in auto-assessment [24].

In the present study, PGXG was taken with 500 mL water immediately before or with a meal. The PGXG was mixed in the water, in juice or mixed into the meal. The mechanism for weight loss in the PGXG per-protocol sub-group in the present study may have been as a result of reducing dietary energy density of the meals with which it was consumed by increasing the fibre and water content of meals while maintaining the volume of food eaten [37,38]. Decreasing dietary energy density has been shown to be useful in long-term weight loss [39].

The detailed dietary feedback from the mFR enabled this research to determine weight loss was possible when PGXG at the recommended dose was consumed and the reduction in the number of eating occasions may be part of the mechanism for this effect. Previous research indicates that a possible mechanism behind the appetite and body weight reduction effects of PGX may be related to circulating gut hormones [30]. Reimer et al. [30] reported increased peptide YY, which can slow gastric emptying and decreased ghrelin (an appetite stimulant) on consumption of PGX. More work is required to confirm the physiological mechanisms controlling these effects of PGX.

The detailed dietary feedback from the mFR enabled this research to more accurately determine food group changes after 12 weeks. The reason the PGXS, and PGXG per-protocol groups did not reduce their weight may be because they did not reduce the number of eating occasions or change their daily intake of the foods and continued to consume the amount and type of food usually eaten. In addition, research by Polidori et al. [40] reports that weight loss leads to increased appetite, and appetite increases by approximately 100 kcal/day per kg of lost weight. The reason fibre such as PGXG helps some individuals in the control of appetite and not others requires more research.

### Limitation

Meals and consumption of test products were self-administered; the possibility of non-compliance could not be avoided. In the current study misreporting of intake may have occurred due to participants not taking images of all food and beverages consumed. In addition, the assessment of food group serves by a trained analyst may not be sensitive enough to detect changes in dietary intake.

## 5. Conclusions

Supplementation with PGX at the recommended dose resulted in a reduction in body weight (kg), BMI (kg/m^2^), reduced frequency of eating and reduced intake of white bread. The weight loss and BMI reduction from baseline to 12 weeks was significantly greater for PGXG at the recommended dose than for the RF treatment. Dietary assessment using the mFR provided detailed information enabling accurate analysis of the number of eating occasions and changes to food group servings per day. Further research on reducing the frequency eating of specific foods, such as junk food is warranted. These results demonstrate the potential benefits of PGX fibre in controlling frequency of eating and in weight loss.

## Figures and Tables

**Table 1:** Serve size in g or mL National Health and Medical Research Council (NHMRC), 2013 [27]. Fibre is 1 serve (g), with 2.5 serves daily minimum recommended dose.

**Grains**				**Vegetable**	**Fruit**	**Meats**			**Dairy**		**Junk Food**		Fibre *	
Bread	Porridge cooked	Muesli/Oats raw	Rice Pasta cooked			Beef Lamb Pork	Chicken	Fish	Milk	Cheese	Meat pie	Donut or Cake	PGXS	PGXG
40	120	30	75–120 (1/2 cup)	75	150	65	80	100	250 mL	40	60	40	3.8	4.4

* Dose determined by study. PGXS = PGX softgel, PGXG = PGX granules.

**Table 2:** Characteristics of all recruited study participants randomised at baseline (*n* = 120) comparing groups.

All Participants	PGXS (*n* = 40)	PGXG (*n* = 40)	RF (*n* = 40)
Men	9	10	9
Women	31	30	31
Mean ± SD			
Age (years)	42.2 ± 16.0	46.5 ± 14.0	43.3 ± 16.8
Height (cm)	167.4 ± 9.1	167.3 ± 9.0	166.4 ± 7.9
Weight (kg)	82.7 ± 16.8	80.9 ± 16.6	81.3 ± 17.7
Waist (cm)	89.8 ± 12.8	90.7 ± 12.1	88.4 ± 14.3
Body Mass Index (BMI, kg/m^2^)	29.4 ± 4.8	28.7 ± 4.4	29.2 ± 4.8
Eating occasions per day	5.4 ± 2.8	6.3 ± 2.0	4.8 ± 2.1
Food group servings (mean daily serves ± SD)			
Fruit (150 g)	0.8 ± 0.8	1.0 ± 1.0	1.1 ± 1.2
Vegetable (75 g)	2.4 ± 1.5	2.6 ± 1.4	2.5 ± 1.1
Grain (cereal) (40 g bread, 75–120 g cooked rice, pasta etc. or 500 kJ)	3.8 ± 1.8	4.3 ± 2.2	4 .0 ± 1.5
Dairy (250 mL milk, 40 g cheese or 500–600 kJ)	1.3 ± 0.9	1.6 ± 0.8	1.3 ± 0.8
Junk food (60 g meat pie or hot chips, 40 g donut or cake)	3.5 ± 1.9	3.1 ± 1.6	3.0 ± 1.7
Meat (65 g meat, 100 g fish or 500–699 kJ)	1.0 ± 0.7	1.6 ± 0.8	1.4 ± 0.7
Alcohol (150 mL)	0.5 ± 0.8	0.4 ± 0.5	0.1 ± 0.2

PGXS = PGX softgel, PGXG = PGX granules, RF = Rice Flour. SD = Standard Deviation.

**Table 3:** Characteristics of study participants randomised at baseline (*n* = 54) comparing subgroups who consumed the recommended dose of fibre supplements.

All Participants	PGXS (*n* = 17)	PGXG (*n* = 18)	RF (*n* = 17)	*p* Value
Body weight (kg)	76.5 ± 15.9	87.7 ± 20.2	78.3 ± 15.0	0.62
BMI (kg/m^2^)	27.2 ± 4.5	28.7 ± 5.2	28.3 ± 5.2	0.64
Waist (cm)	84.8 ± 12.2	89.2 ± 20.4	87.1 ± 13.8	0.59
Eating occasions per day	7.4 ± 2.5	6.0 ± 2.0	5.5 ± 2.6	*p* > 0.05

PGXS = PGX softgel, PGXG = PGX granules, RF = Rice Flour.

**Table 4:** Change in participant characteristics from baseline to week 12 of the interventions of all participants who completed the interventions (intention-to-treat analysis).

All Participants	PGXS (*n* = 32)	PGXG (*n* = 32)	RF (*n* = 19)
Body weight (kg)	0.47 ± 1.85	−0.49 ± 0.34	−0.03 ± 0.58
BMI (kg/m^2^)	0.15 ± 0.65	−0.17 ± 0.13	0.01 ± 0.20
Waist (cm)	−0.17 ± 2.92	**−2.50 ± 0.60 *p* = 0.03**	−1.3 ± 1.0
Eating occasions per day	−0.60 ± 1.5	**−0.82 ± 1.28 *p* = 0.01**	−0.22 ± 1.72
Food group servings (mean daily serves ± SD)			
Fruit (150 g) ^1^	−0.2 ± 0.76	0.08 ± 0.7	−0.18 ± 0.75
Vegetable (75 g) ^1^	−0.07 ± 1.11	−0.34 ± 1.22	−0.23 ± 0.64
Grain (cereal)	0.21 ± 1.73	−0.79 ± 1.66	−0.51 ± 1.23
Dairy	0.11 ± 0.63	−0.22 ± 0.64	0.07 ± 0.35
Junk food	−0.14 ± 2.00	−0.57 ± 1.29	0.28 ± 2.12
Meat	0.08 ± 0.59	0.01 ± 0.79	−0.09 ± 0.78
Alcohol	−0.22 ± 0.73	0.17 ± 0.99	−0.02 ± 0.26
Fibre (3.8–4.4 g) ^1^	1.89 ± 0.91	2.17 ± 0.71	2.35 ± 0.58

Bold values denote significant within treatment effect. PGXS = PGX softgel, PGXG = PGX granules, RF = Rice Flour. ^1^ 1 serve of fruit = 150 g, 1 serve vegetable = 75 g, 1 serve fibre = 3.8 to 4.4 g.

**Table 5:** Change in participant characteristics from baseline to week 12 of the PGXS, PGXG and RF interventions in the subgroup analysis of those who consumed the recommended dose of fibre supplements (per-protocol analysis).

All Participants	PGXS (*n* = 17)	PGXG (*n* = 18)	RF (*n* = 17)
Body weight (kg)	0.22 ± 1.61	**−1.4 ± 0.10 *p* < 0.01**	−0.03 ± 0.58
BMI (kg/m^2^)	0.07 ± 0.59	**−0.5 ± 0.10 *p* < 0.01**	0.01 ± 0.20
Waist (cm)	−1.04 ± 2.28	−1.2 ± 1.00	−1.3 ± 1.0
Eating occasions per day	−1.3 ± 1.9	**−1.4 ± 1.20 *p* < 0.01**	−0.22 ± 1.72
Food group servings (mean daily serves ± SD)			
Fruit (150 g)	−0.43 ± 0.59	**−0.63 ± 0.57 *p* = 0.022**	−0.18 ± 0.75
Vegetable (75 g)	−0.35 ± 0.96	−0.82 ± 1.31	−0.23 ± 0.64
Grain (cereal)	−0.93 ± 1.47	**−1.52 ± 1.84 *p* = 0.019**	−0.51 ± 1.23
Dairy	−0.05 ± 0.56	**−0.59 ± 0.50 *p* = 0.012**	0.07 ± 0.35
Junk food	−0.17 ± 1.48	−0.76 ± 0.85	0.28 ± 2.12
Meat	−0.06 ± 0.62	0.18 ± 0.90	−0.09 ± 0.78
Alcohol	−0.50 ± 0.98	0.11 ± 0.32	−0.02 ± 0.26
Fibre supplement serves (serving size 4.5–5 g)	2.6 ± 0.47	2.82 ± 0.24	2.35 ± 0.58

Bold values denote significant within treatment effect. PGXS = PGX softgel, PGXG= PGX granules, RF = Rice Flour. SD = standard deviation.
