# Erratum: Effect of Fibre Supplementation on Body Weight and Composition, Frequency of Eating and Dietary Choice in Overweight Individuals *Nutrients* 2017, *9*, 149

**DOI:** 10.3390/nu9040409

**Published:** 2017-04-20

**Authors:** Vicky A. Solah, Deborah A. Kerr, Wendy J. Hunt, Stuart K. Johnson, Carol J. Boushey, Edward J. Delp, Xingqiong Meng, Roland J. Gahler, Anthony P. James, Aqif S. Mukhtar, Haelee K. Fenton, Simon Wood

**Affiliations:** 1 School of Public Health, Faculty of Health Sciences, Curtin University, Perth, WA 6845, Australia; d.kerr@curtin.edu.au (D.A.K.); w.hunt@curtin.edu.au (W.J.H.); s.johnson@curtin.edu.au (S.K.J.); T.P.James@curtin.edu.au (A.P.J.); Aqif.Mukhtar@curtin.edu.au (A.S.M.); h.fenton@curtin.edu.au (H.K.F.); simonwood@shaw.ca (S.W.); 2 Epidemiology Program, University of Hawaii Cancer Center, Honolulu, HI 96813, USA; cjboushey@cc.hawaii.edu; 3 Video and Image Processing Laboratory, School of Electrical and Computer Engineering, Purdue University, West Lafayette, IN 47907, USA; ace@ecn.purdue.edu; 4 Flinders Centre for Innovation in Cancer, School of Medicine, Flinders University, Adelaide, SA 5001, Australia; rosie.meng@flinders.edu.au; 5 Factors Group R & D, Burnaby, BC V3N4S9, Canada; rgahler@naturalfactors.com; 6 Curtin Health Innovation Research Institute, Faculty of Health Sciences, Curtin University, Perth, WA 6845, Australia; 7 Centre for Population Health Research, Faculty of Health Sciences, Curtin University, Perth, WA 6845, Australia; 8 InovoBiologic Inc., Calgary, AB Y2N4Y7, Canada; 9 Food, Nutrition and Health Program, University of British Columbia, Vancouver, BC V6T1Z4, Canada

The authors requested the following corrections to their paper [1]. 

In the abstract ‘intention-to-treat’ was replaced with ‘per-protocol analysis’.

In the statistical analysis the ‘intention-to-treat’ reference was removed.

In the results section: The headings of Section 3.1.1 3.2.1 and 3.3.1 were changed to ‘Per-Protocol Analysis’ and the headings of Sections 3.1.2, 3.2.2 and 3.3.2 were changed to read ‘Subgroup Analysis”.

In Table 4 ‘intention-to-treat’ was re-labelled as ‘per-protocol’ and in Table 5 ‘per-protocol’ was removed from the heading. 

In the text of Sections 3.2.1 and 3.3.1, ‘intention-to-treat’ was changed to ‘per-protocol’. In the text of Sections 3.2.2 and 3.3.2 ‘per-protocol’ was changed to ‘subgroup analysis’.

In the discussion section, ‘intention-to-treat’ was changed to ‘per-protocol’ and ‘per-protocol’ was changed to ‘subgroup analysis’.

The authors apologized for any inconvenience caused to the readers by these changes, stating that they do not affect the scientific results. The original manuscript will be modified accordingly.
